# Interventions to reduce readmissions: can complex adaptive system theory explain the heterogeneity in effectiveness? A systematic review

**DOI:** 10.1186/s12913-018-3712-7

**Published:** 2018-11-26

**Authors:** Lauren S. Penney, Musarrat Nahid, Luci K. Leykum, Holly Jordan Lanham, Polly H. Noël, Erin P. Finley, Jacqueline Pugh

**Affiliations:** 1 South Texas Veterans Health Care System, 7400 Merton Minter Blvd, San Antonio, TX 78229 USA; 2 Department of Medicine, The University of Texas Health Science Center San Antonio, 7703 Floyd Curl Drive, San Antonio, TX 78229 USA; 3 Department of Information, Risk and Operations Management, McCombs School of Business, The University of Texas at Austin, 2110 Speedway Stop B6500, Austin, TX 78712-1277 USA; 4 Department of Family & Community Medicine, The University of Texas Health Science Center San Antonio, 7703 Floyd Curl Drive, San Antonio, TX 78229 USA; 5 Department of Psychiatry, The University of Texas Health Science Center San Antonio, 7703 Floyd Curl Drive, San Antonio, TX 78229 USA

**Keywords:** Complex adaptive systems, Hospital readmissions, Care transitions, Health care interventions, Patient education, Self-organization

## Abstract

**Background:**

Successfully transitioning patients from hospital to home is a complex, often uncertain task. Despite significant efforts to improve the effectiveness of care transitions, they remain a challenge across health care systems. The lens of complex adaptive systems (CAS) provides a theoretical approach for studying care transition interventions, with potential implications for intervention effectiveness. The aim of this study is to examine whether care transition interventions that are congruent with the complexity of the processes and conditions they are trying to improve will have better outcomes.

**Methods:**

We identified a convenience sample of high-quality care transition intervention studies included in a care transition synthesis report by Kansagara and colleagues. After excluding studies that did not meet our criteria, we scored each study based on (1) the presence or absence of 5 CAS characteristics (learning, interconnections, self-organization, co-evolution, and emergence), as well as system-level interdependencies (resources and processes) in the intervention design, and (2) scored study readmission-related outcomes for effectiveness.

**Results:**

Forty-four of the 154 reviewed articles met our inclusion criteria; these studies reported on 46 interventions. Nearly all the interventions involved a change in interconnections between people compared with care as usual (96% of interventions), and added resources (98%) and processes (98%). Most contained elements impacting learning (67%) and self-organization (69%). No intervention reflected either co-evolution or emergence. Almost 40% of interventions were rated as effective in terms of impact on hospital readmissions. Chi square testing for an association between outcomes and CAS characteristics was not significant for learning or self-organization, however interventions rated as effective were significantly more likely to have both of these characteristics (78%) than interventions rated as having no effect (32%, *p* = 0.005).

**Conclusions:**

Interventions with components that influenced learning and self-organization were associated with a significant improvement in hospital readmissions-related outcomes. Learning alone might be necessary but not be sufficient for improving transitions. However, building self-organization into the intervention might help people effectively respond to problems and adapt in uncertain situations to reduce the likelihood of readmission.

**Electronic supplementary material:**

The online version of this article (10.1186/s12913-018-3712-7) contains supplementary material, which is available to authorized users.

## Background

Successfully transitioning patients from the hospital to home is a complex task. As hospital readmission rates have been adopted as markers of quality with financial penalties for poor performance, care transition improvement efforts have multiplied.

Over the last 30 years, a variety of care transition interventions have been implemented, including those with differing types and numbers of components; some have proven effective but many have not [1–3]. There is a lack of information about which components or combinations of components are critical to reducing early (30 day) hospital readmissions [4, 5]. We believe that one source of this variability in effectiveness is due to a mismatch between the inherent complexity of care transitions and the health care organizations in which they occur, and the types of interventions being attempted.

Health care organizations have been studied as complex adaptive systems (CAS) for many years [6–8], contributing insights about the importance of interdependencies among different parts of the system, the role of learning, and the impact of local interactions in self-organization (stable but dynamic patterns of interaction among system components), emergence (system level behaviors that cannot be explained by examining the individual components of the system), and co-evolution (patterned changes that take place as a system and its environment react to each other over time) [9]. Using the lens of CAS provides a theoretical approach for describing and understanding care transition interventions.

Effective care transitions from inpatient to outpatient require multiple diverse individuals (providers [inpatient and outpatient, multiple professions, multiple specialties], patients and caregivers) to interact, share their expertise, learn together, and depend on each other to make sense of what is happening in real time to prevent rehospitalization. CAS theory helps us understand both the complexity of care transitions and the difficulty in predicting which processes or approaches will most likely contribute to success. There is a high degree of uncertainty in predicting a patient’s trajectory of recovery after hospitalization, and therefore their specific transitional needs [10].

CAS theory brings focus to the inherent uncertainty in many clinical tasks, and in clinical systems [10]. CAS theory also suggests that for interventions to be successful, they must be congruent with the uncertainties and contextual differences that people face when they deliver care. For example, interventions that rely on a strict application of a new process from one clinical setting to another may not be as successful as one that allows for local adaptation that reflects differences in the ways that people organize themselves across systems. Interventions that foster interconnections between individuals may be more likely to improve communication and support local potential for co-evolution [11], thus increasing implementation success. Moreover, in complex interventions like those required for improving care transitions, the line between intervention and implementation is often blurred [12], as activities aimed at increasing coordination between inpatient and outpatient care teams (intervention strategy) may also function to educate providers about local care transitions resources (implementation strategy) [13].

This paper is the third in a series of articles examining the association between the degree to which interventions to improve complex care delivery activities are consistent with CAS and their degree of effectiveness. In the first of these papers [14], we found type II diabetes intervention effectiveness was positively associated with (a) the number of CAS characteristics inherent in intervention designs and (b) the degree to which the intervention impacted CAS characteristics of interconnections between individuals, and the degree to which the intervention allowed for evolution in its implementation over time. In the second paper [15], congestive heart failure intervention effectiveness was related to (a) the number of CAS characteristics present in the intervention design and (b) the CAS characteristics of learning, self-organization, and co-evolution, again suggesting the need for interventions to adapt and evolve with local patterns of care organization. We hypothesized that observed differences across our two analyses were due to differences in the ways that diabetes and heart failure lead to uncertainty for patients in their daily lives. These differences require emphasis on different CAS characteristics for successful interventions.

This paper extends this work by examining care transition interventions that move patients across complex microsystems from hospital to home. Such transitions are characterized by uncertainty and dynamic change, and frequently involve changes in patients’ functional capacities, requiring them to learn new acute and chronic self-care practices. These transitions also require successful transfer of information between medical providers and between medical providers and informal caregivers.

Our objective is to explore whether care transition intervention designs that reflect the complexity of processes and conditions they are trying to improve are associated with better outcomes. We also aim to identify key characteristics of interventions that are related to better hospital readmissions outcomes. We hypothesize that care transition interventions with elements that are congruent with the nature of CAS will be more likely to have improved readmission outcomes. We do not suggest that interventions were developed with CAS in mind, but that they vary in their degree of congruence with important CAS principles.

## Methods

Because of the extensive literature on care transitions, we used Kansagara et al.’s report [4], “Transitions of Care from Hospital to Home” as our starting point. This report was prepared for the Department of Veterans Affairs Evidence-based Synthesis Program and represented a comprehensive assessment of the literature on care transitions.

Kansagara et al. identified systematic reviews of care transitions, screening them for quality (e.g., the review clearly reported their search criteria, evaluated the internal validity of included trials). Eighty-three systematic reviews met their initial criteria. They further narrowed this group to 17 of the most recent and broadly scoped systematic reviews that fit pre-identified categories. Ten of those systematic reviews focused on different types of care transition interventions (e.g., enhanced access to primary care, telephone-based follow-up) and 7 focused on specific patient populations. All included reviews contained hospital readmissions as a reported outcome, though it may not have been the primary outcome and not all studies included in the reviews reported hospital readmissions as an outcome [4]. This approach provided us a high quality, diverse set of care transition intervention types upon which to test our research question.

### Inclusion and exclusion criteria

As our interest was in care transition interventions that could be applied to a wide variety of patient populations and not unique to a given disease state, we excluded the 7 targeted patient population reviews included in the Kansagara et al. review [4]. Two researchers (JP and LP) reviewed the remaining reviews in Kansagara et al. and determined that one additional review [16] could be excluded based on the review’s inclusion of studies of low quality and its limited scope (i.e. post-operative care after pancreatic surgery), leaving 154 studies from 9 reviews to be assessed for inclusion in our review. One researcher (LP) reviewed the list to exclude articles for which we could not obtain full text, that were not written in English, and that were duplicates of articles found in one of the other eight reviews, yielding 148 unique publications that were screened for inclusion and exclusion criteria (see Table 1).

**Table 1:** Inclusion criteria

Criterion	Definition
Randomized Controlled Trial	The study is a randomized controlled trial.
Readmissions Outcome	The individual article reports hospital readmissions.
Possibility of Readmission	Every subject/participant in the study has the possibility of being readmitted.
Intervention to Improve Care Transitions	The intervention is aimed at improving one or more care transition processes
Adults	Participants in the study are adults >age 18
Superiority Trial	The study is a superiority trial AND includes tests of significance for the readmissions outcome measure(s).

**Fig. 1:**
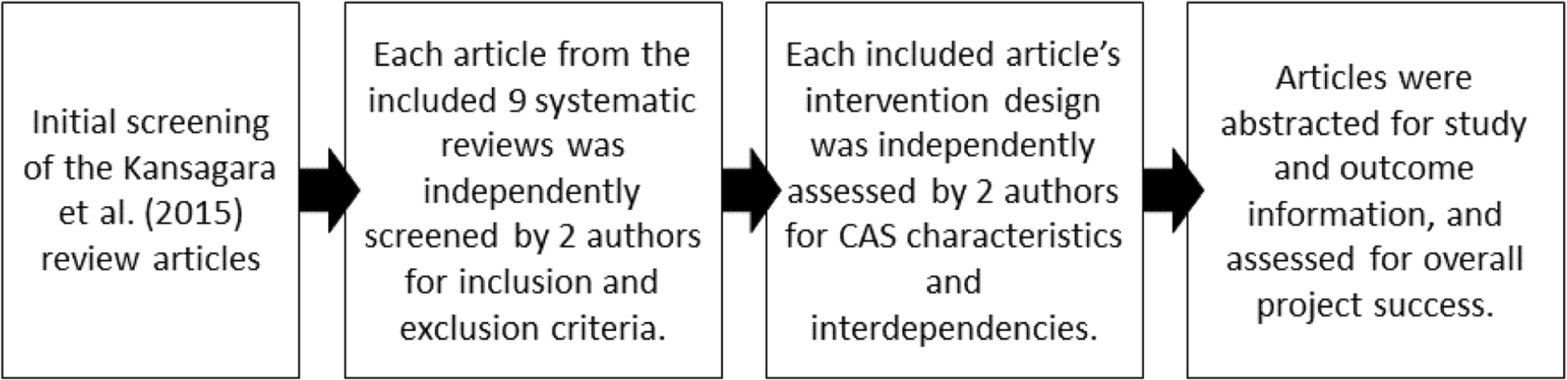
Flow chart of review process. Legend: Steps taken in the initial selection, review, and abstraction of articles

We limited our studies to randomized controlled trials as one proxy of quality (in addition to the quality inclusion criteria in the original reviews). To have a consistent outcome to associate with intervention characteristics, we only included articles that reported hospital readmissions as an outcome. We also excluded studies in which not every participant had the possibility of being readmitted to the hospital (e.g., studies that recruited some participants from the emergency room). We included only studies for which interventions were focused on improving some aspect of care transition processes, as defined by Kansagara et al. [4]. These included processes such as anticipatory discharge planning and care coordination, psychosocial needs assessment, transmitting discharge summaries to outpatient providers, and discharge medication reconciliation. The final inclusion criteria were that study participants be adults and that the study be a superiority trial, with tests of significance performed on the readmission outcome measure(s). Each article was independently reviewed by 2 researchers (see Fig. 1). After scoring for inclusion and exclusion criteria, pairs met to discuss discrepancies. The full research team conferred about and resolved any unresolved discrepancies.

### Assessment of leveraging of characteristics of CAS

As described in our prior studies of diabetes and congestive heart failure [14, 15], interventions were categorized based on the presence or absence of 5 CAS characteristics. For this analysis, we added additional dimensions of complexity to our assessment based on evolution of the CAS literature since our original studies. This work has emphasized the interdependencies between the people (relationships), processes, and infrastructure (affordances) in the system (Table 2). We felt this was particularly important because care transition processes typically cross more than one part of a health care organization. Because relationships were already accounted for in the original CAS characteristic of interconnections, we added processes and affordances. Definitions were drawn from our previous work and refined when the group had scored 5 test articles.

**Table 2:** CAS characteristic and interdependencies’ scoring criteria

	Definition
CAS Characteristic	
Learning	People can and will process information, as well as react to changes in information. Education occurred explicitly or there is a new strategy for information uptake.
Interconnections	Change in pattern of interactions, including nonverbal communication, among agents. Introducing new agents into the system.
Self-organization	Order is created in a system without explicit hierarchical direction. Interventions explicitly allow for modifications, tailoring, adjustments, and negotiations based on patient characteristics, situations, circumstances, and uniqueness of patients, at the level of patients.
Co-evolution	The system and the environment influence each other’s development. Adaptations, additions or changes to the intervention or implementation of the intervention that affect more than one patient typically in response to new information or interim evaluation of intervention.
Emergence	Intervention is leveraging the fact that non-linearities will occur in the system—Specifically plans to look (e.g., scanning, monitoring) for unintended consequences to try to use them to own advantage.
Interdependencies	
Affordances	Something new (e.g., staff, process) was brought to the care transition under focus. New resources have been brought in to change an outcome of interest. This might include new staff but could be old staff doing a new thing (i.e. reallocation of old resources or allocation of new resources).
Processes	Any standard workflow change or work standardization could be in the form of a process checklist.

After the scoring guide was finalized (see Table 2), each article was assigned to be read and independently scored by two researchers. For each intervention described in the included studies, each CAS characteristic was scored as either 0 (absent) or 1 (present). Pairs who had independently scored the studies met to identify and resolve discrepancies in scoring, and to ensure intercoder reliability. Scoring that could not be decided among the pairs was discussed in group meetings and final decisions about scoring were made by the group.

After scoring was completed, pairs also abstracted the articles to document sample size, readmission-related outcome unit(s), readmission-related results, and overall project success. Outcome units were described in terms of the timeframe (e.g., 30 days, 1 year) and the measurement (e.g., incidence of readmissions, length of stay of readmissions). Researchers described results in terms of whether there were statistically significant differences between the study groups on the readmission-related outcome measures. Because of the heterogeneity of outcome measurement, overall intervention success was scored dichotomously as either 0 (not effective) and 1 (effective) as described below in Table 3.

**Table 3:** Scores for intervention effectiveness

Score	Description	Criteria
0	No effect	None of the readmission-related outcomes were significantly better for the intervention group
1	Intervention effective	At least one readmission-related outcome was significantly better in the intervention group

### Analysis

We examined the distribution of CAS characteristics and outcomes among the studies. From this, we identified the CAS characteristics that were most variable in terms of presence or absence in the intervention design (e.g., the CAS characteristic was neither rarely nor nearly always present). We conducted chi squared tests to investigate the significance of the relationship between each of those characteristics and outcomes.

We conducted a second statistical analysis using Fisher’s exact test extension developed by Mehta and Patel [17], to test the relationship between the total number of variable CAS characteristics each intervention had and its effectiveness. Studies were scored with a 0, 1, or 2 based on how many of the CAS characteristics with the most variability were reflected in their intervention design. All statistical analyses were performed using Stata 14 [18].

## Results

### Included studies

**Fig. 2:**
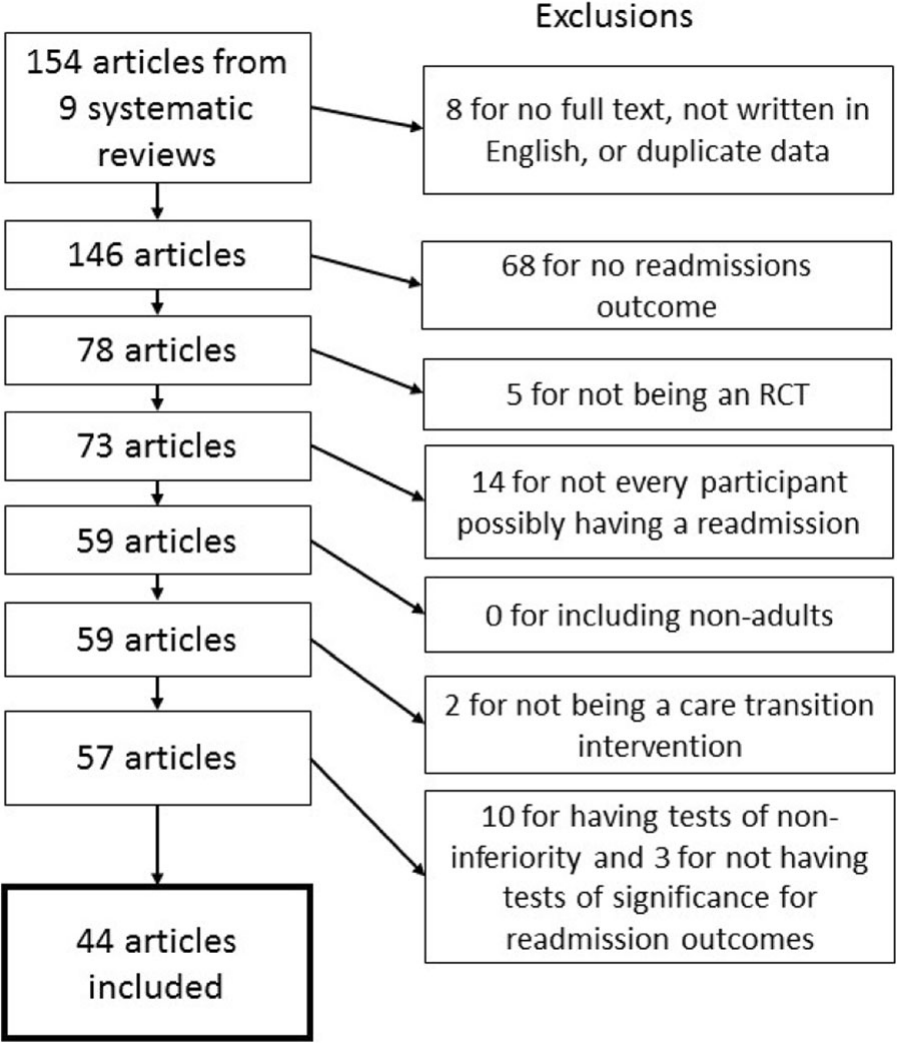
Procedure for selecting articles. Legend: Flow chart depicting details of articles which were excluded from this analysis based on inclusion criteria

We initially reviewed 154 publications from the 9 systematic reviews [19–27] for potential inclusion in our analysis. Eight publications were immediately excluded because we could not obtain full text copies (*n* = 4), the full text was not written in English (*n* = 2), or contained duplicate data from another publication included in the Kansagara review(*n* = 2). After screening full text of the remaining 146 articles, 102 publications were excluded for not meeting inclusion criteria (see Fig. 2). The majority (67%) were excluded for not having a hospital readmissions outcome.

Forty-four publications were included in our analysis [28–71] (see Additional file 1). Studies had a mean sample size of 420 people (range: 24 to 2353). There was heterogeneity in readmissions outcomes, including, percent of patients readmitted within 30 days, duration of all cause rehospitalizations at 180 days, the incidence of unexpected admissions at 5 weeks, and the proportion of participants readmitted at 6 months. The number of readmission-related outcomes reported also varied, ranging from 1 to 18. On average, studies reported 3 readmission related outcomes, however the majority (65%) reported 1 or 2 readmissions-related outcomes.

### Assessment of interventions

Two publications described three armed studies, with two interventions apiece. All other studies described a single intervention. Each of the 46 interventions described in the 44 included publications was scored separately for their use of the five CAS characteristics and two interdependencies (see Additional file 2). The types of interventions reported varied from implementation of new discharge forms, to use of alerts, to multi-pronged interventions that included new post-discharge contacts between patients and healthcare providers. Almost all the interventions involved a change in interconnections from care as usual (96% of interventions), and added affordances (98%) and processes (98%) (see Table 4). The majority (67%) of interventions included learning and 69% allowed for self-organization. No intervention reflected qualities of either co-evolution or emergence, meaning that none allowed for adaptation of the intervention over time, or planned to look for results that could then be used to update the intervention.

**Table 4:** Score distribution for CAS characteristics, interdependencies, and outcomes

	n (percent)	Examples
CAS Characteristic		
Learning	31 (67%)	• Nurses used behavior skill training strategies with patients and encouraged their self-monitoring and use of external cognitive supports. • Nurses provided patients education on such things as vital signs, activities of daily living, coping skills, and signs and symptoms.
Interconnections	44 (96%)	• Advanced practice nurses contacted patients after discharge. • Case managers met with patients after each physician visit.
Self-organization	32 (69%)	• Physicians were alerted if patient values went outside normal range; if deemed necessary, physicians could ask patients to adjust medication use. • Nurses could modify or tailor the frequency of patient follow-up calls based on patient symptoms, knowledge, and needs.
Co-evolution	0 (0%)	(none)
Emergence	0 (0%)	(none)
Interdependencies		
Affordances	45 (98%)	• Patients were provided personal telecare units. • Patients were given discharge forms in one of three languages.
Processes	45 (98%)	• An appointment reminder was mailed to patients 10 days prior to each appointment. • Patient discharge forms were electronically transferred to primary care nurses.
Positive Intervention Effect Reported	18 (39%)	(not applicable)

Sixty-one percent of the interventions included in our analysis did not lead to statistically significant improvements in any reported hospital readmission-related outcomes (i.e., between intervention and control groups). Thirty-nine percent of interventions had at least one readmission-related outcome that was significantly better for the intervention group than the control.

### Learning and self-organization, by outcomes

Because 95% or more of the interventions incorporated the CAS characteristics of interconnections, affordances, and processes, we did not analyze the association of those characteristics with outcomes. Instead, we restricted our analyses to learning and self-organization, which had greater variability in their distribution across interventions (67 and 69%, respectively). Interventions that influenced learning, such as those that taught self-management skills, or allowed for self-organization, such as allowing providers and patients to change frequency of communication based on patient needs, were more likely to report one or more significant readmission outcomes than interventions that did not (see Tables 5 and 6). The association between readmission outcomes and CAS characteristics was not significant (*p* = 0.064 for learning and *p* = 0.104 for self-organization). Although interventions with the properties of self-organization were more likely to have significant outcomes than interventions that lacked self-organization (47% vs. 21%, respectively), this association was not significant.

**Table 5:** Score distribution and chi square test results by outcome for selected CAS characteristics

Selected CAS Characteristic Score	Rating of Intervention Effectiveness, n (row %)		Chi square
	Not Effective (*n* = 28)	Effective (*n* = 18)	
Learning – 0	12 (80%)	3 (20%)	3.42, *p* = 0.064
Learning – 1	16 (52%)	15 (48%)	
Self-Organization – 0	11 (79%)	3 (21%)	2.65, *p* = 0.104
Self-Organization – 1	17 (53%)	15 (47%)

**Table 6:** Score distribution by outcome and combined selected CAS characteristics

Absence (0) or Presence (1) of Selected CAS Characteristics		Rating of Intervention Effectiveness			Fisher’s exact test
Learning	Self-Organization	Not Effective (*n* = 28)	Effective (*n* = 18)	Percent Effective	*p* = 0.005
0	0	4	2	33%	
1	0	7	1	12%	
0	1	8	1	11%	
1	1	9	14	61%	

When scores for learning and self-organization were combined, we identified a significant relationship among the variables. Interventions rated as effective were significantly more likely to have both characteristics of learning and self-organization (61%) than interventions rated as having no effect (39%; *p* = 0.005).

## Discussion

We assessed whether care transition interventions whose design was congruent with CAS characteristics were more likely to report reduction in early readmissions. We found that nearly all the interventions assessed contained elements reflecting the 3 CAS characteristics of interconnections, affordances and processes, and none of the interventions displayed coevolution or emergence. The CAS characteristics that were most inconsistently reflected in our group of included studies were learning and self-organization. When both of these characteristics were present, interventions were associated with significantly fewer readmissions.

These results complement those reported by Leppin et al.’s work, in which early hospital readmission interventions were analyzed using the cumulative complexity model framework [3]. Their review included 42 trials compared to our 46, nine of which we shared in common. At least five of their included trials would have been excluded from ours because they were conducted on surgical patients. Leppin et al.’s analysis identified that interventions that were more complex (defined as involving more people or components), and those that increased patient capacity for self-care, were more likely to be effective. While that framework appears to share some similarities to CAS (e.g. attention to feedback loops, emergence), it focuses on workload-capacity imbalances and patient complexity, and considers intervention complexity in terms of component parts rather than their impact on characteristics of the complex systems in which the interventions are applied [72]. Whereas Leppin et al’s work was directed toward the intervention components, our CAS-grounded work was more focused on the interdependencies among the components in the systems of intervention. It is possible that their findings regarding self-care are related to learning, and that interventions with more components might be more likely to change interdependencies or self-organization. Future work might look at how the two approaches could be synthesized to enhance our understanding of applying complex interventions in complex adaptive systems.

Our findings have some important differences from our previous work examining the relationships between interventions’ congruence with CAS characteristics and outcomes in two complex chronic diseases: diabetes and chronic heart failure. In the case of type 2 diabetes interventions [14], interconnections and co-evolution were positively associated with outcomes. In our present analysis, 96% of the interventions changed interconnections, typically through approaches such as post-discharge calls or novel handoff practices. The fact that most of the interventions were designed to change the way people interacted with each other but were associated with varied results suggests that interconnections alone were not sufficient for improving outcomes. Additionally, in this group of studies, most of the changes in interaction patterns focused on providers (e.g., patient-provider, provider-provider). Changing other types of interconnections (e.g. between patients and their family caregivers) might influence different aspects of care delivery that is highly dependent on patient and family actions.

Similar to our current findings, in our review of chronic heart failure interventions [15], learning, self-organization, and co-evolution were significantly related to intervention effectiveness. In our analysis of care transition interventions, learning alone was not significantly associated with effective outcomes; when combined with self-organization, its association on readmissions was significant. This finding suggests that learning might be necessary to consider when trying to decrease readmissions, but perhaps is not sufficient. Returning home after a hospital stay involves many life changes, not all which can be anticipated and prepared for through patient education. As in the case of interconnections, different types of learning might also need to be distinguished to determine which approaches are more effective.

Care transitions may lead to a higher degree of uncertainty for patients and providers than other aspects of care delivery, including chronic disease management. After hospitalization, patients may experience medical, functional, social, and spiritual changes. While the interventions we studied made changes to the clinical or healthcare system, they frequently did not address the home context to which patients were returning. Learning and self-organization may be particularly important in this situation as they may help patients effectively respond to problems that might arise after they have returned home, reducing their likelihood of readmission. For example, interventions that include an aspect of patient education (like teaching congestive heart failure patients to manage weight and adjust diuretics) may help patients to know what to expect and respond.

One commonality between our studies of diabetes, congestive heart failure, and care transitions is that, in each study, interventions that had an adaptive element were more successful, whether it be through allowing local self-organization among individuals in the system, or recognizing that implementation is an evolutionary process that requires change over time. We found few descriptions of how interventions were modified or adapted over time, and no examples of co-evolution in the interventions we reviewed. This may spring from a desire for intervention fidelity, or typical word count limitations in the medical literature. Our findings across our three studies speak to the importance of the need for adaptive approaches. The complex and dynamic nature of clinical systems, and of patients’ trajectories over time, speaks to the need for adaptive approaches in which individuals can better tailor the intervention and implementation strategy to the ways they have organized care over time. More details of these adaptive approaches (such as detailed protocol papers) should be reported in the literature regarding these interventions. Increased interest in adaptation in the implementation science literature (e.g., [73, 74]) is helping expose the importance of both carefully documenting modifications as they happen and understanding adaptations as important for intervention success.

### Limitations

This project has several limitations. Our assessment of CAS characteristics was limited by the published descriptions of the interventions, which may be inadequate for understanding intervention context and adaptations. In addition, we defined CAS characteristics in broad terms, with each characteristic potentially encompassing an array of diverse intervention practices. Thus, many studies may appear similar in terms of the CAS characteristics, but the specifics of the interventions and the contexts in which they were implemented may contain important differences. For example, our definition of learning is both limited (e.g. interventions had to explicitly describe an educational component or learning, not inclusive of implicit learning that occurs during interpersonal interactions) and possibly overly expansive (e.g. does giving a patient a handout really count as learning?). Future research should consider how best to assess how learning occurs in complex healthcare interventions, particularly in the context of high uncertainty. Our dichotomous assessment of effectiveness was possibly also too generous, as any intervention could be scored as effective if at least one of the reported readmission outcomes was significant. Having a more nuanced classification scheme for study outcomes might have yielded different results.

The breadth of our dataset is both a strength and limitation. The articles reviewed are from a large span of time during which this literature evolved rapidly and expectations for the description of study methodology and intervention details changed significantly. Finally, our starting point was articles identified in a review of reviews, and we relied upon the original reviews to have assessed for methodological quality. Different systematic reviews used different methods for assessing quality. If we had started de novo with a search for all interventions addressing readmissions as an outcome, we may have identified additional studies.

## Conclusion

Transitional care interventions with characteristics of complex adaptive systems, particularly learning and self-organization, are associated with a greater reduction in early readmissions. New study designs allowing for adaptation are needed to better address interventions in the complex clinical systems in which they are implemented.

## Additional file

Additional file 1: Intervention description. Characteristics of included studies of care transition interventions, as described in Penney, et al., Interventions to Reduce Readmissions: Can complex adaptive system theory explain the heterogeneity in effectiveness? A systematic review. (DOCX 45 kb)

Additional file 2: Complexity scoring. Ratings of eligible studies by complex adaptive system characteristics (CAS), interdependencies, and project success, as described in Penney, et al., Interventions to Reduce Readmissions: Can complex adaptive system theory explain the heterogeneity in effectiveness? A systematic review. (DOCX 44 kb)

## Abbreviations

CAS: Complex adaptive systems
