# Supplementary material for: Interventions to reduce readmissions: can complex adaptive system theory explain the heterogeneity in effectiveness? A systematic review
Source: BMC Health Serv Res. 2018 Nov 26;18:894. doi: 10.1186/s12913-018-3712-7 (PMC6260570; doi:10.1186/s12913-018-3712-7)
Supplement: Supplementary file 1 — Intervention description. Characteristics of included studies of care transition interventions, as described in Penney, et al., Interventions to Reduce Readmissions: Can complex adaptive system theory explain the heterogeneity in effectiveness? A systematic review. (DOCX 45 kb) [file 12913_2018_3712_MOESM1_ESM.docx]

Additional File 1: Characteristics of included studies of care transition interventions, as described in Penney, et al, Interventions to Reduce Readmissions: Can complex adaptive system theory explain the heterogeneity in effectiveness? A Systematic Review.

| **Author, Year** | **Sample Size (n)** | **Readmission Outcome Unit of Measurement** | **Outcomes Significantly Different?** |
| --- | --- | --- | --- |
| ***Review:*** Crocker, Crocker, & Greenald, 2012 | | ***Intervention Type: Telephone Follow-Up from Primary Care*** | |
| Balaban, Weissman, Samuel, & Woolhandler, 2008 | 196 | 31-day readmission rate | No |
| Fitzgerald, 1994 | 668 | Number of readmissions per patient per month in VA hospitals for non-elective or for any cause; number of readmission days per patient per month in VA hospitals for non-elective or for any cause; number of readmissions and number of readmission days per patient per month in non-VA hospitals; number of readmissions and number of readmission days per patient per month total | No |
| Smith, Weinberger, Katz, & Moore, 1988 | 1001 | Incidence of readmissions and of readmission days per patient per month for elective, for non-elective, or for all causes (stratified by 3 patient risk levels) | No |
| ***Review:*** Ellis, Whitehead, Robinson, O’Neill, & Langhorne, 2011 | | ***Intervention Type: Comprehensive Geriatric Assessment*** | |
| Asplund et al., 2000 | 413 | Proportion readmitted in 3 months; incidence of readmissions in 3 months | No |
| Kircher et al., 2006 | 315 | Percent with at least one rehospitalization in 3 months; rehospitalization days | Mixed |
| Landefeld, Palmer, Kresevic, Fortinsky, & Kowal, 1995 | 651 | 3-month readmission rate | No |
| Reuben et al., 1995 | 2353 | Incidence of readmissions in 3 months | No |
| Thomas, Brahan, & Haywood, 1993 | 120 | 6-month readmissions | Yes |
| White et al., 1994 | 40 | 30-day readmission rate | No |
| Winograd, 1993 | 197 | Incidence of readmissions in 12 months; hospitalization days in 12 months | No |
| ***Review:*** Huntley et al., 2013 | | ***Intervention Type: Case Management*** | |
| Avlund, Jepsen, Vass, & Lundemark, 2002 | 149 | Proportion readmitted in 3 months | No |
| Lim, Lambert, & Gray, 2003 | 598 | Incidence of unplanned admissions in 6 months; frequency of unplanned admissions in 6 months; hospital days used in 6 months | Mixed |
| Melin & Bygren, 1992 | 249 | Proportion readmitted in 6 months; average days spent readmitted in 6 months | No |
| Naylor et al., 1999 | 363 | Percent readmitted 1 or fewer times and 2 or more times in 24 weeks; incidence of readmissions that were index-hospitalization related, were co-morbidity related, were new health problem related, and were any cause related; time of readmission in 6 weeks and in 6-24 weeks; time spent in hospital all, per patient, and per readmitted patient in 24 weeks | Mixed |
| Nikolaus, 1999 | 545 | Percent rehospitalized in 12 months; length of rehospitalization stay | Mixed |
| ***Review:*** Kwan, Lo, Sampson, & Shojania, 2013 | | ***Intervention Type: Medication Reconciliation*** | |
| Jack, 2009 | 749 | Incidence rate ratio in 30 days | Yes |
| ***Review:*** Mistiaen & Poot, 2006 | | ***Intervention Type: Telephone Follow-Up from Hospital*** | |
| Beckie, 1989 | 74 | Incidence of rehospitalization in 6 weeks | Yes |
| Tranmer & Parry, 2004 | 200 | Incidence of unexpected readmissions | No |
| Bostrom, Caldwell, McGuire, & Everson, 1996 | 1413 | 30-day readmission rate | No |
| Dudas, Bookwalter, Kerr, & Pantilat, 2001 | 221 | 30-day readmission rate | No |
| Jerant, Azari, & Nesbitt, 2001 | 25 | Heart failure-related hospitalizations in 6 months; non-heart failure-related hospitalizations in 6 months; all cause hospitalizations in 6 months | No |
| Jerant, Azari, & Nesbitt, 2001 | 24 | Heart failure-related hospitalizations in 6 months; non-heart failure-related hospitalizations in 6 months; all cause hospitalizations in 6 months | No |
| ***Review:*** Pandor et al., 2013 | | ***Intervention Type: Remote Monitoring*** | |
| Angermann et al., 2012 | 715 | Percent with at least one rehospitalization in 180 days; percent rehospitalized for heart failure, for revascularizaton, for pacemaker or ICD, or for nonfatal cardiovascular event in 180 days; mean rehospitalizations in 180 days; mean cardiovascular rehospitalizations in 180 days | No |
| Antonicelli et al., 2008 | 57 | Recurrent hospital admissions in 12-months | Yes |
| Barth, 2001 | 24 | Heart failure-related readmissions in 2 months | No |
| Capomolla et al., 2004 | 133 | Incidence of hospitalization for heart failure, for other cause, and for cardiac cause in 10 +/- 6 months (stratified by 3 patient risk levels) | Mixed |
| Chaudhry et al., 2010 | 1653 | 180-day all-cause readmission and heart failure related readmission incidence; number of days in the hospital; number of readmissions | No |
| Cleland, Louis, Rigby, Janssens, & Balk, 2005 | 253 | Days in hospital for all-cause readmissions in 240 days; days in hospital for heart failure in 240 days; duration of readmission for all-cause in 240 days; duration of readmission for heart failure in 240 days | No |
| Cleland, Louis, Rigby, Janssens, & Balk, 2005 | 258 | Days in hospital for all-cause readmissions in 240 days; days in hospital for heart failure in 240 days; duration of readmission for all-cause in 240 days; duration of readmission for heart failure in 240 days | No |
| Dar et al., 2009 | 182 | Duration of all-cause hospitalization in 180 days; duration of heart failure related hospitalizations in 180 days | No |
| DeBusk et al., 2004 | 462 | Time to first heart failure-related hospitalization in 180 days; to first all-cause rehospitalization in 180 days | No |
| Dendale et al., 2012 | 160 | Incidence of heart failure-related readmissions per patient; number of all-cause readmissions | No |
| Goldberg et al., 2003 | 280 | 180-day hospital readmission rate; 180-day heart failure readmission rate | No |
| Kulshreshtha, Kvedar, Goyal, Halpern, & Watson, 2010 | 150 | 6-month all-cause readmission rate; 6-month heart failure-related readmission rate | No |
| Laramee, Levinsky, Sargent, Ross, & Callas, 2003 | 287 | 90-day all-cause readmission rate; readmission cause; incidence of heart failure-related readmissions; length of stay; cumulative hospital days; days to first readmission | Mixed |
| Rainville, 1999 | 34 | 1-year heart failure related readmission rate | Yes |
| Riegel et al., 2002 | 358 | 3- and 6-month heart failure-related and all-cause hospitalization rates; 3- and 6-month heart failure-related and all-cause readmission rates; 3-month and 6-month heart failure-related and all-cause hospital days; 3- and 6-month inpatient heart failure-related costs; mean time to rehospitalization in 6 months; 6-month incidence of multiple readmissions | Mixed |
| Scherr et al., 2009 | 108 | Hospitalization for heart failure exacerbation | Mixed |
| Tsuyuki et al., 2004 | 276 | 6-month all-cause readmission rate; percent with at least 1 all-cause hospital readmission in 6 months; 6-month cardiovascular-related readmission rate; percent with at least 1 cardiovascular-related readmission | No |
| Wakefield et al., 2008 | 148 | 12-month readmission rate; time to readmission | Mixed |
| Woodend et al., 2008 | 249 | 3-month and 1-year readmission rate for angina patients and for heart failure patients; 3-month and 1-year all-cause readmission rate | Mixed |
| ***Review:*** Shepperd et al., 2013 |  | ***Intervention Type: Discharge Planning*** | |
| Evans & Hendricks, 1993 | 835 | 30- and 90-day readmission rate; average duration of readmission in 30 days and in 90 days | Mixed |
| Naylor, 1994 | 276 | 2-, 2-6, and 2-12 week readmission rate for medical DRG and for surgical DRG; time to first readmission | Mixed |
| Nazareth et al., 2001 | 362 | 3- and 6-month readmission rate | No |
| Shaw, Mackie, & Sharkie, 2000 | 97 | Readmission rate | No |
| Weinberger, Oddone, & Henderson, 1996 | 1396 | Readmission rate | Mixed |
