# Supplementary material for: Interventions to reduce readmissions: can complex adaptive system theory explain the heterogeneity in effectiveness? A systematic review
Source: BMC Health Serv Res. 2018 Nov 26;18:894. doi: 10.1186/s12913-018-3712-7 (PMC6260570; doi:10.1186/s12913-018-3712-7)
Supplement: Supplementary file 2 — Complexity scoring. Ratings of eligible studies by complex adaptive system characteristics (CAS), interdependencies, and project success, as described in Penney, et al., Interventions to Reduce Readmissions: Can complex adaptive system theory explain the heterogeneity in effectiveness? A systematic review. (DOCX 44 kb) [file 12913_2018_3712_MOESM2_ESM.docx]

Additional File 2: Scoring of included studies by complex adaptive system characteristics (CAS), interdependencies, and intervention success, as described in Penney, et al, Interventions to Reduce Readmissions: Can complex adaptive system theory explain the heterogeneity in effectiveness? A systematic review.

| **Author, Year** | **Agents Who Learn (Y/N)** | **Interconnections (Y/N)** | **Self-Organization (Y/N)** | **Co-Evolution (Y/N)** | **Emergence**  **(Y/N)** | **Affordances**  **(Y/N)** | **Processes**  **(Y/N)** | **Project success/ effectiveness**  **(Positive, Mixed, Negative)** |
| --- | --- | --- | --- | --- | --- | --- | --- | --- |
| ***Review:*** Crocker, Crocker, & Greenald, 2012 | | | | ***Intervention Type: Telephone Follow-Up from Primary Care*** | | | | |
| Balaban, Weissman, Samuel, & Woolhandler, 2008 | Y | Y | Y | N | N | Y | Y | Negative |
| Fitzgerald, 1994 | Y | Y | Y | N | N | Y | Y | Negative |
| Smith, Weinberger, Katz, & Moore, 1988 | Y | Y | Y | N | N | Y | Y | Negative |
| ***Review:*** Ellis, Whitehead, Robinson, O’Neill, & Langhorne, 2011 | | | | ***Intervention Type: Comprehensive Geriatric Assessment*** | | | | |
| Asplund et al., 2000 | Y | Y | N | N | N | Y | N | Negative |
| Kircher et al., 2006 | Y | Y | Y | N | N | Y | Y | Mixed |
| Landefeld, Palmer, Kresevic, Fortinsky, & Kowal, 1995 | N | Y | Y | N | N | Y | Y | Negative |
| Reuben et al., 1995 | N | Y | N | N | N | Y | Y | Negative |
| Thomas, Brahan, & Haywood, 1993 | N | Y | N | N | N | Y | Y | Positive |
| White et al., 1994 | Y | Y | N | N | N | Y | Y | Negative |
| Winograd, 1993 | Y | Y | N | N | N | Y | Y | Negative |
| ***Review:*** Huntley et al., 2013 | | | | ***Intervention Type: Case Management*** | | | | |
| Avlund, Jepsen, Vass, & Lundemark, 2002 | N | Y | Y | N | N | Y | Y | Negative |
| Lim, Lambert, & Gray, 2003 | N | Y | Y | N | N | Y | Y | Mixed |
| Melin & Bygren, 1992 | N | Y | Y | N | N | Y | Y | Negative |
| Naylor et al., 1999 | Y | Y | Y | N | N | Y | Y | Mixed |
| Nikolaus, 1999 | Y | Y | Y | N | N | Y | Y | Mixed |
| ***Review:*** Kwan, Lo, Sampson, & Shojania, 2013 | | | | ***Intervention Type: Medication Reconciliation*** | | | | |
| Jack, 2009 | Y | Y | Y | N | N | Y | Y | Positive |
| ***Review:*** Mistiaen & Poot, 2006 | | | | ***Intervention Type: Telephone Follow-Up from Hospital*** | | | | |
| Beckie, 1989 | Y | Y | N | N | N | Y | Y | Positive |
| Tranmer & Parry, 2004 | N | Y | Y | N | N | Y | Y | Negative |
| Bostrom, Caldwell, McGuire, & Everson, 1996 | Y | Y | N | N | N | Y | Y | Negative |
| Dudas, Bookwalter, Kerr, & Pantilat, 2001 | N | N | N | N | N | Y | Y | Negative |
| Jerant, Azari, & Nesbitt, 2001 | Y | Y | Y | N | N | Y | Y | Negative |
| Jerant, Azari, & Nesbitt, 2001 | Y | Y | Y | N | N | Y | Y | Negative |
| ***Review:*** Pandor et al., 2013 | | | | ***Intervention Type: Remote Monitoring*** | | | | |
| Angermann et al., 2012 | Y | Y | Y | N | N | Y | Y | Negative |
| Antonicelli et al., 2008 | Y | Y | Y | N | N | Y | Y | Positive |
| Barth, 2001 | Y | Y | N | N | N | Y | Y | Negative |
| Capomolla et al., 2004 | Y | Y | Y | N | N | Y | Y | Mixed |
| Chaudhry et al., 2010 | N | Y | N | N | N | Y | Y | Negative |
| Cleland, Louis, Rigby, Janssens, & Balk, 2005 | N | Y | Y | N | N | Y | Y | Negative |
| Cleland, Louis, Rigby, Janssens, & Balk, 2005 | N | Y | N | N | N | Y | Y | Negative |
| Dar et al., 2009 | Y | Y | Y | N | N | Y | Y | Negative |
| DeBusk et al., 2004 | Y | Y | Y | N | N | Y | Y | Negative |
| Dendale et al., 2012 | N | Y | Y | N | N | Y | Y | Negative |
| Goldberg et al., 2003 | N | Y | Y | N | N | Y | Y | Negative |
| Kulshreshtha, Kvedar, Goyal, Halpern, & Watson, 2010 | N | Y | Y | N | N | Y | Y | Negative |
| Laramee, Levinsky, Sargent, Ross, & Callas, 2003 | Y | Y | Y | N | N | Y | Y | Mixed |
| Rainville, 1999 | Y | Y | Y | N | N | Y | Y | Positive |
| Riegel et al., 2002 | Y | Y | Y | N | N | Y | Y | Mixed |
| Scherr et al., 2009 | Y | Y | Y | N | N | Y | Y | Mixed |
| Tsuyuki et al., 2004 | Y | Y | N | N | N | Y | Y | Negative |
| Wakefield et al., 2008 | Y | Y | Y | N | N | Y | Y | Mixed |
| Woodend et al., 2008 | Y | Y | Y | N | N | Y | Y | Mixed |
| ***Review:*** Shepperd et al., 2013 | | | | ***Intervention Type: Discharge Planning*** | | | | |
| Evans & Hendricks, 1993 | N | N | N | N | N | N | Y | Mixed |
| Naylor, 1994 | Y | Y | Y | N | N | Y | Y | Mixed |
| Nazareth et al., 2001 | Y | Y | Y | N | N | Y | Y | Negative |
| Shaw, Mackie, & Sharkie, 2000 | Y | Y | N | N | N | Y | Y | Negative |
| Weinberger, Oddone, & Henderson, 1996 | Y | Y | Y | N | N | Y | Y | Mixed |
